# Gender difference in the perceived cause of fall leading to fracture and its potentially contributing factors among older adults

**DOI:** 10.5249/jivr.v15i2.1788

**Published:** 2023-07

**Authors:** Mahdieh Ardaneh, Mohammad Fararouei, Jafar Hassanzadeh

**Affiliations:** ^*a*^ Epidemiology Department, School of Health, Tehran University of Medical Sciences, Tehran, Iran.; ^*b*^ HIV/AIDs Research Center, Shiraz University of Medical Sciences, Shiraz, Iran.; ^*c*^ Research Center for Health Sciences, Institute of Health, School of Health, Shiraz University of Medical Sciences, Shiraz, Iran.

**Keywords:** Falling, Older age, Fracture, Risk factor, Gender difference

## Abstract

**Background:**

The present study aimed to investigate gender difference in the perceived cause (intrinsic or extrinsic) of falls leading to fracture (FLF) and its association with selected social, health, and environmental factors.

**Methods:**

All patients aged 60 years or older who were admitted to two referral hospitals due to FLF from August 1, 2018, to the end of May 2019, were included in the study. An interview-administered questionnaire was used to collect the required data from 300 participants (136 men and 164 women).

**Results:**

When compared to men, women were less physical active, were less smoker, had lower education, had more vision problems, used more sedatives, and were more satisfied with their life (P less than 0.05 for all). No statistical difference was observed between men and women about the perceived cause of Fall.

**Conclusions:**

Although women and men were the same in the perceived cause of fall, they had considerably riskier lifestyles and lower health status. These factors include education, vision condition, physical activity, occupation, and taking sleeping pills. On the other hand, men were more smoker and alcohol user.

## Introduction

The population of older adults is raising dramatically due to the recent improvements in social and health services. In fact, the global population of older adults (60 years old or over) has passed 900 million and it is estimated that the number will reach two billion by 2050. This figure would be bigger than the size of the population of children.^1,2^ This major population shift is altering the major causes of human morbidity and mortality (e.g. from infectious to chronic) and bringing about several new challenges to governments and health authorities.^3^ In Iran, like many other countries, the issue of the aging population has raised major concerns about new challenges to the public health and social care. Based on the results of a census in 2016, more than 7.5 million people (about 9.3% of the Iranian population) were over 60 years of age.^4^ It is also predicted that within the next decade, the population of the older adults in Iran reaches 10.5 million, a number equivalent to about 10.7 percent of the country’s total population.^4,5^

The accidental injuries resulting from falling are the leading causes of disability and death among older people.^6^ This put financial pressure on not only the affected individuals and their families but also the health and social services.^7^ This is because, the treatment of fractures in older people is more expensive, less effective, and takes a longer time.^8^ In addition, bone fracture and its long-lasting consequences among older people cause a variety of psycho-social impairments such as emotional and social dependency, depression, and major reduction in patient’s social relationships and quality of life.^9^ In that regard, the prevention of falls among older adults is an important goal for public health. As a primary but essential step in preventing falling among older people, we need to clearly define its key features (e.g. who, when, where, and how).^10^ Gender is one of the most important key factors in health which is almost always considered in the epidemiology and prevention of human disorders. Measuring differences between men and women in the scenarios that lead to falls is very helpful in designing effective prevention strategies.^11^ The findings of studies on different aspects of falling and the proposed mechanism of their combined actions stimulated vigorous discussions.^12^ It is partly because falling is an exceptionally multidimensional outcome driven by many demographic, behavioral, health, social, and environmental factors.^13^ However, irrespective of the patient’s gender, most studies have focused on only limited aspects of the above factors,^12^ and that knowing these aspects, effective approaches to fracture prevention in older adults are to be established.^14^

The aims of the present study were, first, to investigate the main social, health, and environmental gender-specific features of FLF to see if different approaches should be applied to prevent falling among men and women and second, to investigate the gender-related falling circumstances (perceived cause, time, and place of fall) among the study population.

## Methods 

This is the second phase of a study on falls leading to fracture among older adults. A full description of the methods is provided before.^13^ In summary, this study recruited all eligible older patients who were admitted to Chamran and Ragaei hospitals due to fall-related bone fractures in Shiraz, the capital of Fars province. The hospitals are referral orthopedic centers in the Southern part of Iran. Totally, 320 patients were invited to participate in the study, of which only 0.07% (n=20) did not agree to participate. The study followed the COREQ checklist.

Data collection: A structured interview-administered questionnaire was used to collect the required data. To define the reliability of the questionnaire, a pilot study was conducted on 20 participants who were interviewed twice with a two-week interval.^15^ Based on the results of test-retest analysis, the questionnaire was defined as adequately reliable (Cronbach's alpha= 0.75).^16^

The questionnaire consisted of questions regarding the demographic status of the patients such as age (year), gender, marital status, education, current job, and participant’s perceived economic status. The participants also reported their latest measured height and weight (all participants had their height and weight measured at health centers by their family nurses). In addition, several questions were asked regarding the housing status and living conditions of the participants. The participants were also asked to report how satisfied they are with their life (on a scale of 0 to 10, 0 meaning they are not satisfied at all and 10 meaning they are completely satisfied with their life), what was the duration of their day and night sleep (hours per day and night) and what was the average daily duration of physical activity (i.e. duration of walking, doing housework or other activities and exercise). The participants were also asked to report any history of falling in the past 5 years and the situation that the recent fall happened (i.e., the time, place, and cause of the fall from the patients’ point of view). In addition, the participants were asked if they had any visual impairment that they were aware of. All participants were asked to report the perceived cause of the recent fall when answering the following question: “What is your perception on the cause of the recent falling accident”. The perceived causes of fall (the interested outcome) are divided into two groups defined as extrinsic (slipped, uneven surface, height) or intrinsic (imbalance, vertigo, visual impairment). Details of the interview and the visual acuity test is provided before.^13^ A trained and experienced health nurse interviewed the patients and conducted the visual test. 

**Inclusion and exclusion criteria: **All patients were over 60 years of age (the defined age for older people in Iran), were able to walk before the accident (with or without aid) and were admitted to one of the above-mentioned hospitals during the study period with at least one fracture due to the accident. Patients were excluded if they were not well enough to consciously answer the interviewer’s questions, were blind or deaf. Verbal consent was obtained from the participants since a significant number of patients were illiterate.

**Sampling and statistical analysis: **All eligible patients who were admitted to the hospitals from September 2018 until the May 2019 and were willing to participate were interviewed (n=300). Using ClinCalc website,^17^ (by setting type 1 error at 0.05 and type 2 error at 0.2 (i.e., 80% power) a post-hoc power calculation suggested that the sample size was adequate to detect a significant association between the perceived cause of fall and gender (the key factor in our study) with an OR as small as two. Basic statistical analysis was conducted using contingency tables and measures of central tendency as well as student t, chi-square, and Fisher exact tests. To present the results in a more informative way, the distributions of the study variables are stratified by gender. Multiple logistic regression was applied to measure the association between the type of perceived cause of fall, i.e., extrinsic (slipped, uneven surface, height) or intrinsic (imbalance, vertigo, visual impairment) with other study variables. To do the analysis, SPSS version 22 (IBM Corp., Armonk, N.Y., USA) was used.

## Results

The patients were mostly women (54.7%) and illiterate (57.2%). For both genders, the largest number of patients were between 60-69 years of age. Table 1 shows the distribution of the demographic variables by gender. When compared to men, women were mostly widowed and less educated (P<0.05 for both). Table 2 presents the health-related conditions of the patients by gender. Accordingly, women reported less physical activity, more vision problems, less tobacco, alcohol, sedatives, and hypnotics use (P<0.05 for all). On the other hand, women reported to be more satisfaction with their life (P<0.05). About falling history, 24.3% of men and 37.2% of women experienced at least one fall during the past 5 years (p<0.05). According to the results from Table 3 , compared to women, men were more commonly wearing footwears at the time of fall (80.9% and 74.4%, respectively, P>0.05). Both genders reported “vertigo and imbalance” (33.1% for men and 35.2% for women, p>0.05) and slipping (33.1% for men and 33.3% for women, p>0.05) as the main perceived causes of fall.

**Table 1:** Gender difference in the distribution of quantitative and qualitative variables regarding demographic factors among the partici-pants.

Variable	Category/Scale	Male (n=136)n (%)	Female (n=164)n (%)	P-value*
**Age (year)**	60-69	71(52.20)	80(48.80)	0.374
	70-79	19(14.00)	33(20.10)	
	80≤	46(33.80)	51(31.10)	
**Marriage**	Single, divorced and widowed	20(14.70)	87(53.00)	<0.001
	Living with spouse	116(85.30)	77(47.00)	
**Education**	Illiterate	60(44.10)	112(68.30)	<0.001
	Primary	35(25.70)	36(22.00)	
	Secondary	12(8.80)	7(4.30)	
	Diploma or higher	29(21.30)	9(5.50)	
**Occupation**	Housewife or retired	105(77.20)	158(96.30)	<0.001
	Employed	31(22.80)	6(3.70)	
**Income**	Inadequate	88(64.70)	95(57.90)	0.451
	Relatively adequate	42(30.90)	62(37.80)	
	Adequate	6(4.40)	7(4.30)	

*P-values are based on Chi-Square or independent samples t-test

**Table 2:** Gender difference in the distribution of quantitative and qualitative health related variables among participants.

Variable	Category/Scale	Male (136) Mean±S DOr n (%)	Female Mean±SD Or n (%)	P-value*
**Life satisfaction**	Score (0-10)	4.81±2.68	5.03±3.11	0.619
**Sleep hours per night**	Hours	7.16±2.24	6.85±2.30	0.231
**Sleep hours per day**	Hours	0.65±1.08	0.47±0.93	0.137
**Vision problem**	Yes	72(52.94)	92(56.10)	0.001**
	No	64(47.06)	72(43.90)	
**Physical activity**	Hours per day	1.30±1.77	0.62±0.84	<0.001
**Shopping**	Myself	70(51.47)	66(40.24)	0.033
	Other	66(48.53)	98(59.76)	
**Fall in the past 5 years**	Yes	33(24.30)	61(37.20)	0.016
	No	103(75.70)	103(62.80)	
**BMI**	Kg/m2	23.42±4.05	24.22±4.48	0.109
**Alcohol use**	Yes	10(7.40)	0(0.00)	0.001**
	No	126(92.60)	164(100.00)	
**Tobacco use**	Yes	66(48.50)	55(33.50)	0.008
	No	70(51.50)	109(66.50)	
**History of vertigo**	Yes	50(36.76)	76(46.34)	0.066
	No	86(63.23)	88(53.66)	
**Taking sleeping pills**	Yes	14(10.29)	37(22.56)	0.008
	No	122(89.70)	127(77.44)	

*P-values are based on Chi-Square or independent samples t-test; ** P-values are based on Fisher exact test.

**Table 3:** Falling related factors by gender among the participants.

Variable	Category	Male (n=136) Frequency(%)	Female (n=164) Frequency (%)	P-value*
**Time of fall**	7-12am	61(44.90)	56(34.10)	0.167
	1pm-6pm	34(25.00)	49(29.90)	
	7pm-6am	41(31.10)	59(36.36)	
**Place of fall**	Kitchen, toilet and bathroom	33(24.30)	50(30.50)	0.078
	Room	32(23.50)	46(28.00)	
	Yard	34(25.00)	43(26.20)	
	Street	37(27.20)	25(15.20)	
**Perceived cause of fall**	Extrinsic (slipped, uneven surface, height)	84(60.00)	91(56.20)	0.60
	Intrinsic (imbalance, vertigo, visual impairment)	56(40.00)	71(43.80)	
**Footwear**	Yes	110(80.90)	122(74.40)	0.181
	No	26(19.10)	42(25.60)	

*P-values are based on Chi-Square test

Based on the outcome of the multiple logistic regression analysis selecting the type of perceived causes of fall as extrinsic (slipped, uneven surface, height) or intrinsic (imbalance, vertigo, visual impairment) as the interested outcome, we observed significant associations between the type of perceived cause of fall and several study variables (Table 4). Accordingly, participants were less affected by extrinsic causes if they were taking sleeping pills or sedatives regularly (ORyes/no =0.45, 95%CI=0.21-0.92, p=0.030) and suffering from vertigo (ORyes/no =0.36, 95%CI=0.19-0.65, p<0.001). On the other hand, those with vision problems (ORyes/no =1.88, 95%CI=1.07-3.38, p=0.031) and those who go shopping (ORmyself /other=2.32, 95%CI=1.32-4.14, p=0.004) were more affected by extrinsic rather than intrinsic causes. No association was observed between gender and the type of perceived cause of fall.

**Table 4:** The association of study variables and perceived extrinsic vs. intrinsic causes of fall among older age participants.

Variable	Category/Scale	OR extrinsic/intrinsic*	95% CI	P-value*
**Gender**	Men	Ref	--	--
	Women	1.25	0.72-2.18	0.423
**Sleeping medicine**	No	Ref	--	--
	Yes	0.45	0.21-0.92	0.030
**Vision problem**	No	Ref	--	--
	Yes	1.88	1.07-3.38	0.031
**History of vertigo**	No	Ref	--	--
	Yes	0.36	0.19-0.65	<0.001
**Place of fall**	Bathroom and toilet	Ref	--	--
	Yard	0.81	0.38-1.75	0.601
	Street	1.20	0.52-2.82	0.426
	Room	0.29	0.14-0.62	0.001
	Kitchen	3.18	0.83-16.05	0.116
**Shopping**	Other	Ref	--	--
	Myself	2.32	1.32-4.14	0.004

* Based on multiple logistic regression analysis on extrinsic (slipped, uneven surface, height) versus intrinsic (imbalance, vertigo, visual impairment) perceived causes of fall; the baseline model included: age (year), marriage, education, occupation, income, life satisfaction, sleep hours per night, sleep hours per day, vision acuity, physical activity, fall in the past 5 years, BMI, alcohol use, tobacco use, time of fall, place of fall, and footwear.

## Discussion

According to the study results, patients were predominantly female and history of fall during the past five years was considerably common in both genders. Women and men were different in many aspects of social, demographic, and health-related factors that were potentially related to FLF. The high incidence of fall during the past five years among the study population seems relatively low when compared to the results of a Turkish study in 2016 suggesting that about %28.3 of the older people had a history of fall during the last year.^18^ Other studies also reported a higher proportion of falling history among women compared to men. For example, two studies in America and Sweden suggested that falls among women is more common than men.^19,20^ Table 5 compares the characteristics of fall in our study with other studies worldwide.

**Table 5:** Comparing the results of our article with other articles published around the world from 2017 to 2021.

Country (study)	Most common cause of fall (%)	Most common place of fall (%)	female to Male ratio (age range years)
Iran (current study)	Imbalance	Kitchen toilet, and bathroom	1.21 (≥60)
Turkey(Ozturk, T.C., et al.,)^30^	Stumbling	Room	2.35 (≥65)
India(A. Joseph and J. Muliyil)^31^	difficulty in walking	Open field	1.14(>70)
Malaysia(L. J. WS, et al)^32^	Imbalance	Room	1.73 (≥60)
Greece (Tsellou, M., et al)^33^	--	Outdoor	0.79 (45-96)
southern sri lanka (Gamage, N., et al)^34^	Vision impairment	Stairs	1.40 (≥65)

According to the results of the present study, women were less physically active when compared to men. The results suggested that both genders experienced injurious falls at about the same age. About economic status, no significant difference was observed between the gender of the participants with a significant number of the patients reported inadequate income. In addition, most of the participants were either illiterate or had primary education (mostly among women). A study in Helsinki in 2019 reported that 66.0% of men and 58.5% of women who experienced an injurious fall had lower socio-economic status.^21^ In accordance with the results of the present study, a study in Europe in 2016 suggested that the level of education in most of the older people with injurious falls is low.^22^ The design of the present study prevents us from making any causal inference from the observed results, however, as other researchers noticed, older people with lower socio-economic status may have less access to a safe environment and quality housing.^23^

Based on what was reported by the participants, about 85.7% of the falls occurred at home (including yard) with a slightly lower proportion in men. Women experienced fall most frequently in the kitchen, bath, or toilet. The results of the present study are partly in accordance with the findings of other researchers. As an example, a study in India in 2016, suggested that 87.5% of falls occurred indoors.^24^ Another study in the North-east of Iran in 2017, suggested that 53.3% of the falls occurred indoors.^25^ However, a study on older adults with age-related macular degeneration suggested that most falls occurred outside the home during the daytime activities.^26^ Our findings may suggest that either Iranian older women prefer to spend less of their time outside home unless it was necessary (e.g., shopping) or they have a less safe housing environment.

The results of our study also suggested a significant association between the place of fall and the reported cause of the accident (extrinsic or intrinsic). Accordingly, falls in the room were more likely due to intrinsic causes (vertigo, imbalance, visual impairment). The results also suggested that falls happened mostly in the bathroom, toilet, or kitchen. Similarly, a study in Turkey in 2016 suggested that toilets and bathrooms are the most common places of falling.^18^ About the perceived cause of fall, extrinsic causes (slipping and uneven surface) were more common in men. Whereas, among women, intrinsic factors were the predominant causes of FLF. A study in India in 2016 reported vertigo and imbalance as the predominant cause of fall among Indian older adults.^24^ Again, the relatively large proportion of falls due to unleveled surface and slipping in our study may suggest unsafe housing and environment for Iranian older adults.

Our study also suggested that a history of vertigo and uncorrected vision impairment were common among the participants, and both were also associated with the perceived cause of fall. Accordingly, poor visual acuity raises the likelihood of FLF due to extrinsic factors whereas, falls due to intrinsic factors were more likely to occur among those with a history of vertigo. These results are somehow in accordance with what was reported by other researchers. A published review suggested that vision acuity, contrast sensitivity, glare sensitivity, and visual field size are significantly associated with imbalance, an intrinsic condition that is an important risk factor for falling.^27^ Some also reported that eye diseases such as cataracts and glaucoma are also associated with the risk of fall.^28^ Similarly, a study in 2018 on American people over 65 years of age suggested that sight correction and treatment of vertigo can reduce the risk of falling.^29^

Another interesting result of the current study was the observed significant difference between the two genders in taking sleeping pills. Accordingly, taking sleeping pills among women was more common (more than twice) than men. Taking sleeping pills was also significantly associated with the perceived cause of FLF as those taking pills were more likely to fall due to intrinsic factors. No study reported such an association.

At the time of falling, most patients (especially men) were wearing footwears. Foot wearing, however, was not associated with the reported cause of fall. We also reported a significant association between the perceived cause of the fall (extrinsic) and shopping. The result suggests unsafe streets and outdoor environments for Iranian older adults even in big cities. Again, no study on the subject was found to report.

**Strengths and limitations: **The study participants are from a relatively representative sample of older people with FLF in the community. The participants reported reasons and conditions of falls from their point of view, resulting in better picture of the interaction between the cause of fall and the accident’s key features. However, the study focused on older people whose falling had resulted in severe injuries, requiring hospital admission. As a result, our sample is only a small part of a much wider population of older people who fall. In addition, the descriptive nature of our study prevents us from making any causal inferences about the findings.

**Implementations: **Visual correction was found to be a potentially important intervention as it seems to interact with the key factors of falling such as place, and cause of fall. Making home and streets safer and adequate lighting of indoor and outdoor environments for older people are to be considered in any interventional program. Studies on appropriate footwear for different places are highly recommended.

## Conclusion

Our study revealed more detailed information about FLF among older adults. Unlike the previous studies, we investigated the interaction between key features of falling-related factors (i.e., visual acuity, type of foot wearing, and place) and the perceived cause of fall among men and women. Selecting severe types of falling makes the outcome more effective when planning interventional programs to reduce falling-related disabilities among older adults. We found no association between gender and perceived cause of fall, visual acuity, and place of fall. However, women and men were substantially different in several other important falling related factors (e.g., foot wearing, history of fall, and physical activity). As a result, to be more effective, the falling preventive approaches are to be adjusted for gender.

**Abbreviations**

FLF: Falls leading to fracture.

OR: Odds ratio

CI: Confidence interval

Ref: reference group

**Declarations**

**Ethics approval and consent to participate.**

The participants were assured that their information is used for research purposes only. Because of the illiteracy of a considerable number of the patients, verbal consent was obtained from the participants. The study protocol was reviewed and approved by the ethical committee of Shiraz University of Medical Sciences.

**Acknowledgment**

The authors would like to extend thanks to Shiraz University of Medical Sciences for its support in implementing the project.
